# Energy Expenditure Estimation in Children, Adolescents and Adults by Using a Respiratory Magnetometer Plethysmography System and a Deep Learning Model

**DOI:** 10.3390/nu14194190

**Published:** 2022-10-08

**Authors:** Fenfen Zhou, Xiaojian Yin, Rui Hu, Aya Houssein, Steven Gastinger, Brice Martin, Shanshan Li, Jacques Prioux

**Affiliations:** 1 Sino-French Joint Research Center of Sport Science, College of Physical Education and Health, East China Normal University, Shanghai 200241, China; 2 Département Sciences du Sport et Éducation Physique, Ecole Normale Supérieure de Rennes, 35170 Bruz, France; 3 Laboratoire Mouvement—Sport—Santé (EA 7404), Université de Rennes 2, 35170 Bruz, France; 4 Department of Sports Science and Physical Education, The Chinese University of Hong Kong, Hong Kong 999077, China; 5 Department of Economics and Management, Jianghai Polytechnic College, Yangzhou 225101, China

**Keywords:** energy expenditure, age, respiratory magnetometer plethysmography, physical activities, temporal convolutional networks model

## Abstract

Purpose: Energy expenditure is a key parameter in quantifying physical activity. Traditional methods are limited because they are expensive and cumbersome. Additional portable and cheaper devices are developed to estimate energy expenditure to overcome this problem. It is essential to verify the accuracy of these devices. This study aims to validate the accuracy of energy expenditure estimation by a respiratory magnetometer plethysmography system in children, adolescents and adults using a deep learning model. Methods: Twenty-three healthy subjects in three groups (nine adults (A), eight post-pubertal (PP) males and six pubertal (P) females) first sat or stood for six minutes and then performed a maximal graded test on a bicycle ergometer until exhaustion. We measured energy expenditure, oxygen uptake, ventilatory thresholds 1 and 2 and maximal oxygen uptake. The respiratory magnetometer plethysmography system measured four chest and abdomen distances using magnetometers sensors. We trained the models to predict energy expenditure based on the temporal convolutional networks model. Results: The respiratory magnetometer plethysmography system provided accurate energy expenditure estimation in groups A (R^2^ = 0.98), PP (R^2^ = 0.98) and P (R^2^ = 0.97). The temporal convolutional networks model efficiently estimates energy expenditure under sitting, standing and high levels of exercise intensities. Conclusion: Our results proved the respiratory magnetometer plethysmography system’s effectiveness in estimating energy expenditure for different age populations across various intensities of physical activity.

## 1. Introduction

Physical activity (PA) is defined as any bodily movement produced by the contraction of skeletal muscles that leads to an increase in energy expenditure (EE) above the resting level of an individual [1]. EE is consequently a key parameter in quantifying PA. PA and increasing EE are associated with reduced morbidity and mortality in many chronic diseases, including cardiovascular disease, diabetes mellitus and some forms of cancer [2,3,4,5].

Several tools exist to measure or estimate EE. Reference techniques such as direct calorimetry, indirect calorimetry (IC) and doubly labeled water (DLW) provide the most accurate measurements of EE. However, these methods are limited due to the need for specific equipment and human expertise and the high cost [1]. To overcome these problems, researchers have attempted to develop portable devices that are lightweight and less expensive for estimating EE. Among these devices, accelerometers are increasingly more popular and are often used to predict EE [6] due to their low cost, data simplicity and non-invasive character [7]. However, accelerometers, generally located at the hip, cannot detect arm movements or, for example, exertion during the lifting or pushing of objects. This external work could represent a considerable component of the subject’s lifestyle [8]. Physiological variables, such as heart rate (HR), are also used for estimating EE [9]. Nevertheless, HR could be increased by emotions such as anxiety, a rise in body temperature, or as a postexercise response lag without an evident associated increase in EE [10]. To estimate free-living EE, another physiological variable can be used. Indeed, Durnin et al. [11] and Ford et al. [12] have shown that minute ventilation ($\dot{V}$_E_) can also be used as an index of EE estimation. Recent technological advances to estimate $\dot{V}$_E_ from wearable sensors have encouraged researchers to use this physiological variable to estimate EE [13]. Among these technologies, the respiratory magnetometer plethysmography (RMP) system has been accepted as a reliable method for estimating $\dot{V}$_E_ [14,15,16,17]. However, earlier studies only focused on the adult population. 

Yet, there are significant differences between children and adults in terms of anthropometric, anatomical and respiratory characteristics. Size influences the ventilatory response [18]. The dimensions of the rib cage vary with age during growth [19,20,21]. Respiratory muscle strength increases during growth [22]. Finally, several authors have shown that ventilatory behavior in healthy or pathological adult subjects is complex and compatible with chaotic dynamics [23], particularly by privileging the study of tidal volume (V_T_) using the respiratory plethysmography by inductance (RIP) method [24,25]. Small et al. [26] have shown that ventilatory behavior in children was also complex and chaotic. Furthermore, despite more limited information on EE in children compared to adults, there are also some key differences between children and adults. Children have a higher basal metabolic rate compared to adults [27]. As children attain maturity, there is a gradual decrease in EE with increasing age. Finally, the changes in EE appear to be related most closely to changes in body composition that occur around the time of puberty [28].

Considering the previous differences according to age, the aim of this study was to validate the RMP system for estimating EE in children, adolescents and adults under resting and exercise conditions. We hypothesized that the RMP system can accurately estimate EE regardless of the age of the subjects.

## 2. Materials and Methods

### 2.1. Ethical Approval and Subjects

The experimental protocol was approved by the committee for protecting the people of SUD-EST IV of French 1 (CPPID-RCB-2019-A03303–54). Twenty-three healthy volunteers participated in the study. They were divided into three groups: a group of adults (A) (four males and five females) aged 28.11 ± 2.93 years, eight post-pubertal (PP) males aged 14.75 ± 0.71 years and a group of six pubertal (P) females aged 11.67 ± 0.52 years. All subjects were healthy and presented no pulmonary or heart diseases. Their ages and anthropometric characteristics are displayed in Table 1. The volunteers and their parents provided written consent after being informed of the study’s procedures and objectives.

### 2.2. Protocol

#### 2.2.1. Study Design

**Figure 1:**
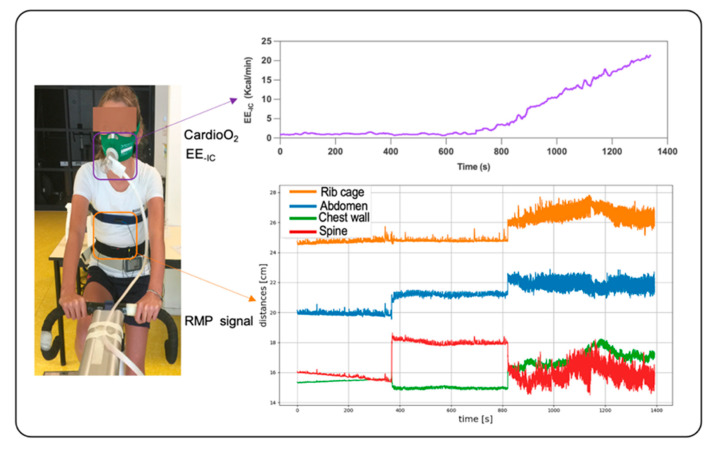
Subjects equipped with a cardioscope, RMP and gas exchange measurement systems. EE_-IC_: energy expenditure measured from indirect calorimetry.

A physician performed a clinical examination and an electrocardiogram upon the subjects’ arrival in the laboratory. Anthropometric measurements were taken, and the pubertal stages of children and adolescents were assessed. Subjects were equipped with a cardioscope, RMP and gas exchange measurement systems (CardioO_2_) (Figure 1). 

**Figure 2:**
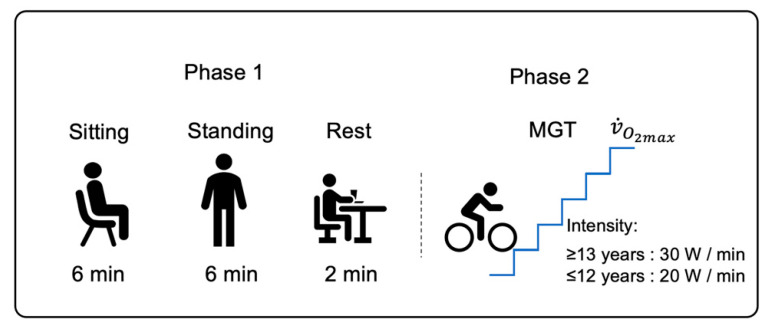
Study design. MGT: maximal graded test; $\dot{V}$O_2max_: maximal oxygen uptake.

Then, the subjects sat or stood for six minutes (Figure 2—Phase 1). During two minutes of rest, we adjusted the subjects’ position on the cycle ergometer, and they began the maximal graded test (MGT) (Figure 2—Phase 2). The testing protocol was conducted in a silent atmosphere to minimize breathing disturbances.

#### 2.2.2. Maximal Graded Test

Each subject was asked to perform an MGT while seated on a cycle ergometer (Excalibur Sport 925909, The Netherlands). An electrocardiogram signal was monitored continuously using a cardioscope (Mortara X12+, Milwaukee, WI, USA), and a continuous progressive protocol was used. The protocol differed according to the subjects’ age [29]: for children ≤12 years old, the exercise intensity was increased by steps of 20 W.min^−1^; for children ≥13 years old, exercise intensity was increased by steps of 30 W.min^−1^. $\dot{V}$O_2max_ was achieved when three of the following criteria were satisfied: (1) a steady HR at a value close to the theoretical maximal HR (HR_max_ = 220−age); (2) a respiratory exchange ratio (R) greater than 1.1; (3) a constant O_2_ despite the increased exercise intensity; (4) the inability of a subject to maintain a pedaling rate of 50 rpm [29].

#### 2.2.3. Ventilatory Thresholds

**Figure 3:**
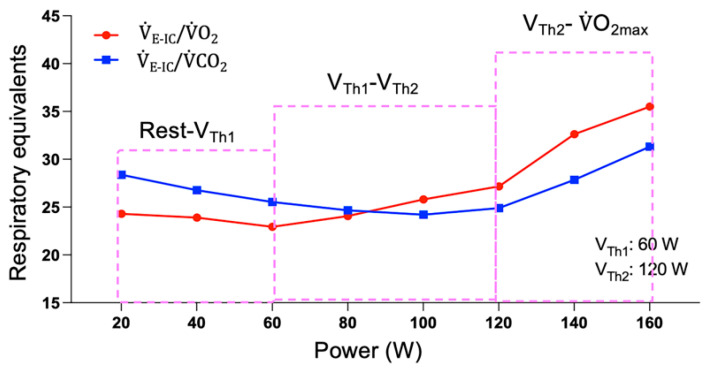
An example of determining the three levels of intensity during the maximal graded test. V_Th1_: first ventilatory threshold; V_Th2_: second ventilatory threshold; $\dot{V}$_E_/$\dot{V}$O_2_: respiratory equivalent in oxygen; $\dot{V}$_E_/$\dot{V}$CO_2_: respiratory equivalent in carbon dioxide; $\dot{V}$O_2max_: maximal oxygen uptake.

Ventilatory responses differ according to exercise intensity [30]. To determine the first ventilatory threshold (V_Th1_) during MGT, we used the respiratory equivalents method [31], in which V_Th1_ corresponds to the point at which the respiratory equivalent in oxygen ($\dot{V}$_E-IC_/$\dot{V}$O_2_) begins to increase while the respiratory equivalent in carbon dioxide ($\dot{V}$_E-IC_/$\dot{V}$CO_2_) remains stable. The second ventilatory threshold (V_Th2_) corresponds to the point at which $\dot{V}$_E-IC_/$\dot{V}$O_2_ continues to increase while $\dot{V}$_E-IC_/$\dot{V}$CO_2_ begins to increase (Figure 3). Two observers determined V_Th1_ and V_Th2_ visually and independently. 

#### 2.2.4. Stage of Development

The same physician assessed the degree of pubertal maturation according to pubic hair growth (Tanner and Whitehouse scale) [32]. This classification divided adolescents into pubertal (stages II and III) and PP (stages IV and V).

#### 2.2.5. Anthropometric Characteristics

The same researcher measured the height and total body mass according to the technical recommendations of the International Biology Program [33].

### 2.3. Measurement Systems

#### 2.3.1. Respiratory Gas Exchange and Heart Rate Measurements

Breath-by-breath measurements of gas exchange were made using an indirect calorimetry system, the Ultima CardioO_2_ (Medical Graphics, St Paul, Minnesota, USA). Before each test, the Ultima CardioO_2_ was calibrated according to manufacturers’ guidelines. EE_-IC_, oxygen uptake ($\dot{V}$O_2_), carbon dioxide production ($\dot{V}$CO_2_) and $\dot{V}$_E_ were measured and displayed continuously on the computer screen. Data were collected during each breath and transferred to a PC for live display. The recorded data were saved in the internal database of the Ultima CardioO_2_ for precise performance analysis after the test. HR was recorded using the cardioscope connected with the Ultima CardioO_2_.

#### 2.3.2. RMP System

**Figure 4:**
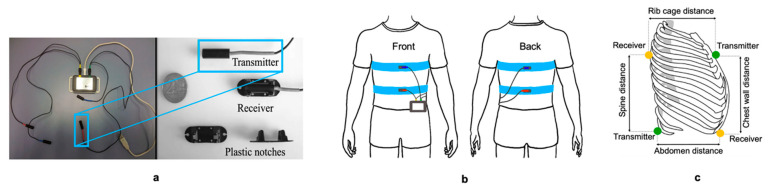
(**a**): Overview of the RMP system; (**b**): position of transmitters and receivers on the subject using elastic belts; (**c**): distances measured using the RMP system.

The RMP system (Nomics s.a., Liège Science Park, Belgium) had been described by Dumond et al. [16] and Houssein et al. [17]. This device is lightweight (80 g) and portable (85 mm × 55 mm × 16 mm), with two pairs of electromagnetic coils connected to a recorder (battery) box. Each pair coil consists of a transmitter and receiver (Figure 4a). A resonant frequency circuit in the transmitter generates a magnetic field. The magnetic field is detected by the receiver and converted into a signal. The distance (cm) between the transmitter and receiver is calculated using magnetic field intensity. Figure 4b shows the specific position of the sensor transmitters and receivers on the subject using elastic belts. The first receiver is placed in a posterior position on the spine, opposite the first transmitter. The second receiver is placed in the anterior position on the midline of the abdomen, just above the umbilicus, opposite the second transmitter. The RMP system measures four distances (Figure 4c): the anteroposterior displacement (cm) of the rib cage (RC) and the abdomen (AB) and the axial displacements of the chest wall (CW) and the spine (SP). The data were recorded at a sampling rate of 15 Hz. The device recorded and stored signals in its internal memory. The memory capacity is 16 MB, corresponding to approximately 100 h of recording. The RMP system is powered by a lithium battery that can be recharged using a universal serial bus connection. When the battery is fully charged, the autonomy of this method is sufficient to ensure several nights of recording (minimum 60 h). The recordings were transferred from the device to the computer using APIOS software.

### 2.4. Data Processing

#### 2.4.1. Method for Determining Three Levels of Intensity during the Maximal Graded Test

According to V_Th1_ and V_Th2_, we divided MGT into three intensity ranges (Figure 3). The first range was between rest and V_Th1_, the second was between V_Th1_ and V_Th2_ and the third was between V_Th2_ and $\dot{V}$O_2max_.

#### 2.4.2. Window Segmentation and Feature Extraction

Considering that the IC system measured respiratory parameters breath-by-breath, according to the time of each breathing cycle, the data were recorded individually. Normally, each breathing cycle consists of 2–4 s [34]. Therefore, to cover at least one breathing cycle, the slide window was set to five seconds to calculate the mean value. The overlap of the window is 80%. The Temporal convolutional network (TCN) model extracted features automatically.

#### 2.4.3. Network Architecture and Training Model

##### Network Architecture

**Figure 5:**
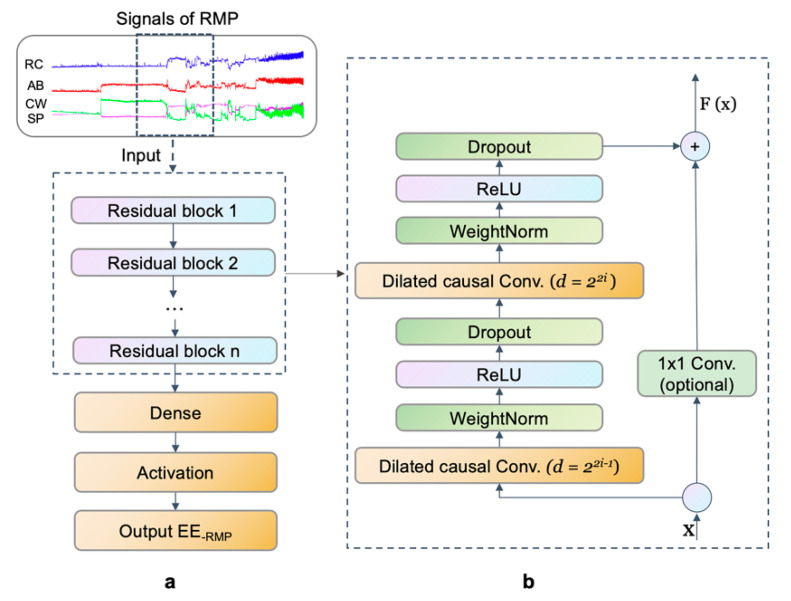
Architecture of the temporal convolutional network model. (**a**) Process, (**b**) an example of the residual block. EE_-RMP_: Energy expenditure measured from respiratory magnetometer plethysmography. Signals of RMP including four distances: RC, AB, CW, and SP. RC: rib cage; AB: abdomen; CW: chest wall; SP: spine.

As a DL model, the TCN model contains residua blocks comprising dilated causal convolutional neural networks, residual connection and others, illustrated in Figure 5a. The aim was to predict EE at time t, denoted by EE, _t_. More precisely, given a prediction model M: $x^{T}\to y$, EE_,t_ is predicted by historical temporal features,$\;x_{i}\in {\mathbb{R}}^{n}$, only up to the current time. W represents the effective history; the prediction equation is defined as follows:

(1) $$\hat{{\mathrm{EE}}_{,t}\;}=M\;\left(x_{t}{,x}_{t-1}{,\ldots ,x}_{t-w}\right)$$

In our TCN model, feature extraction is carried out in sequential layers with kernel dilation for the multi-scale aggregation of input data [35,36]. The process is shown in Figure 5a. By adding the input x into the residual block x through identity mapping (or 1 × 1 convolution in the first residual block if the number of channels is not equal to the residual function shape), the residual function F(x) was derived. An example of the residual block is shown in Figure 5b. Inspired by “ResNet”, the first three dilations were pooled into a single residual block in models with an odd number of dilations [37]. After that, “ResNet” learns the function across two or three layers. Accordingly, the kernel size *k* and the number of exponential dilations n were used to determine the (causal) receptive field:

(2) $$RF=1+\left(k-1\right)\left(2^{n}-1\right),$$

The features at the last time point t were extracted using the splice function for use in the regression network. Thus, a fully connected dense layer and linear activation were used to predict EE_,t_. In our TCN model, we set sixty-four filters, five dilations and two residual blocks, and the kernel size was set at 3 s. The system was implemented using TensorFlow (2.0.0) [38].

##### Training Model

The three groups’ results comprised 19865 samples: 7950 samples in group A, 7040 samples in group PP and 4875 samples in group P. The data of each population group were split into three datasets, including a training set, a validation set and a test set. A total of 55% of the data was used for training, 20% was used for validation and 25% was allocated for testing. The training dataset was used during the learning process. The test dataset was used only to test the ability to generalize the trained model. Five-fold cross-validation was used to evaluate the model’s performance with the validation set to diminish the over-filtering. The network of the learning rate was 0.001, the batch size was 1024 and the epochs were 80. The data in the CSV file were processed and analyzed in Python (Version 3.7.11 for macOS, Stichting Mathematisch Centrum Inc., Guido van Rossum, The Netherlands).

### 2.5. Statistical Analyses

The number of windows required to obtain a robust model which will make it possible to achieve a given percentage of accuracy in the estimation of EE has been analyzed. To achieve an estimation percentage of 95% with a margin of error of 2% and a confidence level of 95%, a minimum of 457 windows were required for the analysis (n = z^2^ × p (1 – p)/m^2^; z = 1.96, *p* = 0.05 and m = 0.02). The analysis of only 5 min of recording per intensity level and the partition of these 5 min into 5 s windows to calculate the features allow for the obtention of a total of 120 analyzable windows (5 × 12 × 5 = 120) for a single subject, i.e., 720 for 6 subjects, 960 for 8 subjects and 1080 for 9 subjects. The Kolmogorov–Smirnov test was used to test the normal distribution of the data. In the absence of normal distribution, the data were log-transformed before analysis. To assess the performance of the models in estimating EE, we compare the mean value of different intensities between EE_-IC_ and EE_-RMP_. Graphical representations of EE_-RMP_ versus EE_-IC_ were plotted with their associated regression line. Bland–Altman analysis with corresponding 95% limits of agreement was used to assess and visualize differences between EE_-IC_ and EE_-RMP_. The values associated with EE_-RMP_ versus EE_-IC_ were presented on a graph. The paired *t*-test was conducted to compare the differences between EE_-IC_ and EE_-RMP_. *p* < 0.05 was considered statistically significant. The data analysis was conducted in GraphPad Prism (Version 9.3.0 for macOS, Graphpad Inc., San Diego, CA, USA). The most common parameters used for the model performance assessment were the root mean square error (RMSE) and the coefficient of determination (R^2^).

## 3. Results

### 3.1. Model Performance

**Figure 6:**
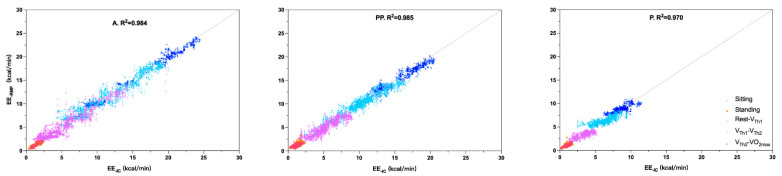
Estimated (EE_-RMP_) and measured (EE_-IC_) energy expenditure individual values for each group. A: adult; PP: post-pubertal; P: pubertal; EE_-IC_: energy expenditure measured from indirect calorimetry; EE_-RMP_: energy expenditure estimated from respiratory magnetometer plethysmography; V_Th1_: first ventilatory threshold; V_Th2_: second ventilatory threshold; $\dot{V}$O_2max_: maximal oxygen uptake.

**Figure 7:**
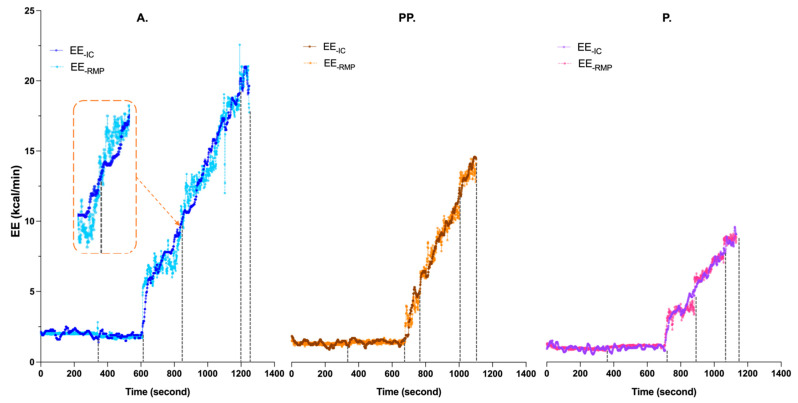
An example of EE_-RMP_ with the TCN model in the three groups. Each dashed line is the dividing line for each intensity level. A: adult; PP: post-pubertal; P: pubertal; EE_-IC_: energy expenditure measured from indirect calorimetry; EE_-RMP_: energy expenditure estimated from respiratory magnetometer plethysmography.

**Figure 8:**
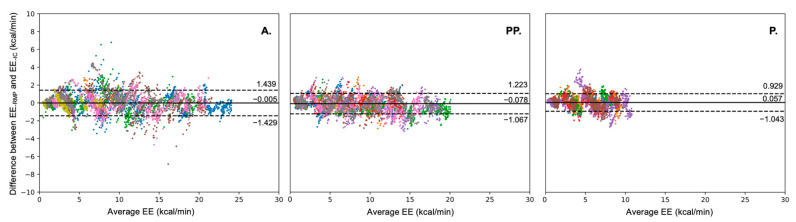
Difference between EE_-RMP_ and EE_-IC_ in the three groups. A: adult; PP: post-pubertal; P: pubertal; EE_-IC_: energy expenditure measured from indirect calorimetry; EE_-RMP_: energy expenditure estimated from respiratory magnetometer plethysmography; The mean difference (bias) between EE_-RMP_ and EE_-IC_ is depicted by the solid line, and the 95% confidence intervals (±2SD) are depicted by the dashed lines. Average EE = (EE_-RMP_ + EE_-IC_)/2. Each color represents an individual. Each color represents data from a unique participant.

To evaluate the performance of EE estimation for different intensities, we developed the TCN model with data from the MGT. With this model, we estimated EE and $\dot{V}$O_2_ in the three groups. In each group, the performance of the TCN model is high, with low RMSE values and high R^2^ values (Table 2). The highest R^2^ (0.985) value was observed in group PP, and the lowest R^2^ (0.970) value was observed in group P. RMSE decreases with age from 0.739 kcal/min in group A to 0.612 kcal/min in group PP and 0.489 kcal/min in group P. The TCN model also shows good results in the $\dot{V}$O_2_ estimation. The level of R^2^ is extremely close in the three groups, ranging from 0.972 in group P to 0.979 in group PP. The RMSE values are extremely close between groups A (2.094 mL/min/kg) and PP (2.041 mL/min/kg). The RMSE value for group P is slightly higher (2.244 mL/min/kg). The predicted value of EE obtained with the TCN model was plotted in Figure 6. Our results logically showed that the maximal value of EE increased with age. Model estimation timelines are shown in Figure 7. EE_-RMP_ could illustrate that our result shows EE_-IC_ in all intensities. However, our results show the differences between EE_-IC_ and EE_-RMP_, especially at the beginning of each intensity-increasing phase. As an example, as shown inside the orange dashed line plotted in Figure 7 group A, when the intensity “Rest-V_Th1_” changes to the next intensity (V_Th1_-V_Th2_), the estimated error increases as well. In the Bland–Altman plots (Figure 8), more than 95% of the points lie within the limits of agreement intervals in the three groups. However, underestimation was observed in groups A and PP (biases were −0.005 and −0.078, respectively), while overestimation was observed in P (the bias was 0.057). 

### 3.2. Comparison of EE_-IC_ and EE_-RMP_ at Different Intensity Levels

To validate the estimating accuracy of EE_-RMP_, we compared EE_-IC_ and EE_-RMP_ for the five intensities and for the three groups. Our results (Table 3) showed, for each group, that EE increased with increasing intensity. When comparing EE_-RMP_ to EE_-IC_, our results showed significantly (*p* < 0.05) higher values of EE_-IC_ for the “standing” intensity in group A and significantly (*p* < 0.001) higher values of EE_-RMP_ for the “standing” intensity in group P. Our results also showed significantly (*p* < 0.01) higher values of EE_-IC_ for the “Rest-V_Th1_” intensity in group PP. No other significant differences were observed.

## 4. Discussion

Our study was based on that of Gastinger et al. [13], who already validated the RMP system for estimating EE in adults under resting and moderate exercise conditions using linear regression. Our objective was to validate the RMP system for assessing EE in children, adolescents and adults under resting and different exercise intensity conditions using a DL model.

Direct and indirect calorimetry are both valuable and accurate techniques for assessing EE under laboratory and field conditions [39]. The direct calorimetry technique is the gold standard for assessing EE in healthy and diseased subjects [39]. The subject is placed in a thermally isolated chamber, and the total heat loss from the body is recorded accurately and measured precisely [40]. Due to the complexity of the equipment required, it is the most expensive and least practical way to measure EE [41]. The indirect calorimetry is the most widely employed method to measure $\dot{V}$CO_2_ and $\dot{V}$O_2_ [40]. In some situations, this indirect calorimetry technique has advantages compared to the direct calorimetry technique. For instance, metabolism increases in direct proportion to exercise intensity during exercise, and $\dot{V}$O_2_ reaches a steady state within one to two minutes [42]. The indirect calorimetry can respond faster for assessing the oxidative heat. In contrast, the direct calorimetry is a more delayed response because it is not paralleled by a similarly rapid increase in heat loss [43]. However, when using the indirect calorimetry, it is crucial to attach the face mask properly and calibrate the equipment before collecting data in order to obtain high-quality EE data. In addition, the battery of these indirect techniques consists of two or three hours and is also expensive. DLW is also a gold standard technique for measuring EE, with low error rates over seven to fourteen days [41]. However, this technique cannot record minute-by-minute information like indirect calorimetry. Thus, the DLW technique cannot be used in some exercises that monitor EE per minute. Additionally, the high price of DLW is also a limitation for researchers. Therefore, research is increasingly focusing on lightweight, low-cost portable devices for EE estimation.

Using a lightweight and portable device, our results show that EE values increased with increasing intensity for each group, regardless of the measurement system used (Table 3, Figure 6), which seems to agree with the literature. In adults aged 27 ± 5 years old, Westerterp et al. [44] monitored total EE (TEE) using the DLW technique. The PA level (PAL) was obtained as the TEE/REE ratio, with REE corresponding to resting EE (REE). PAL increased from 1.52 (lying, sitting and standing) to 2.04 (housework, gymnastics and sport). Chowdhury [45] measured EE, in adults aged 27 ± 6 years old using indirect calorimetry (Cosmed’s K_4_b_2_) during different types of 10 min PAs. EE increased with increasing exercise intensity from 1.51 ± 0.39 kcal/min (typing) to 4.03 ± 1.07 kcal/min (sweeping), 4.12 ± 0.97 kcal/min (walking at 4 km/h), 7.44 ± 1.42 kcal/min (cycling at 75 W for females and 100 W for males) and 9.90 ± 2.01 kcal/min (running at 8.4 km/h). Adamakis et al. [46] measured EE, using indirect calorimetry, in healthy boys (aged 15.00 ± 2.29 years old) and girls (aged 16.55 ± 1.70 years old). Adolescents participated in overground walking (5.89 ± 0.62 km/h) and submaximal running (11.25 ± 1.52 km/h), 1200 m each. Boys expended 62.94 ± 12.93 kcal for walking and 74.60 ± 16.20 kcal for running, while girls expended 58.82 ± 10.34 kcal for walking and 69.51 ± 10.89 kcal for running. Steenbock et al. [47], used accelerometers to estimate EE in children aged 3 to 6 years old during vigorous intensities of PAs. EE increased with increasing exercise intensity from drawing (4.2 ± 0.9 kJ·min^−1^) to regular walking (8.2 ± 2.4 kJ·min^−1^) and tricycling (13.4 ± 3.8 kJ·min^−1^). Lee et al. [48] measured EE, using indirect calorimetry (Cosmed’s K_4_b_2_), in children aged 10- to 13-years-old, who performed a series of PAs of different intensities. The EE values measured in boys and girls gradually increased from resting (boys: 1.12 ± 0.38 kcal/min; girls: 1.06 ± 0.27 kcal/min) to running at 4 km/h (boys: 3.66 ± 1.08 kcal/min; girls: 3.68 ± 1.14 kcal/min) and 8 km/h (boys: 7.46 ± 1.80 kcal/min; girls: 7.99 ± 2.04 kcal/min).

Table 3 shows very similar EE values between groups A and PP for each intensity. Lopez et al. [49], using indirect calorimetry, observed similar trends for measured EE between adolescents (15.3 ± 0.7 years old) and adults (37.7 ± 9.8 years old) during sitting (1.6 vs. 1.5 METs), standing (1.8 vs. 1.6 METs), walking slow (4.0 vs. 3.5 METs) and running (8.2 vs. 10.8 METs) PAs. Table 3 and Figure 6 also show that EE values seem lower in group P than in the other two groups, regardless of the level of intensity and the measurement system used. Ekelund et al. [50] showed that, during different PAs, the PAEE measured by accelerometry was significantly higher in adolescents aged 17.6 ± 1.5 years old than it was in children aged 9.6 ± 0.3 years old, and Bitar et al. [51] showed a significant increase in exercise-related EE in pubertal boys compared to prepubertal boys. Therefore, EE values are lower in children than they are in adolescents, both in boys and girls [52]. Indeed, even though children expend more energy per kilogram of body weight than adults in performing PAs of a given intensity, their lower body weight leads to a smaller overall EE compared to older subjects [53].

Based on the relationship between EE and $\dot{V}$_E_ [11,12,13], Gastinger et al. [13] validated the RMP system in estimating EE in adults during various PAs (sitting, standing and walking at 4, 5, 6 km/h). They showed that EE_-IC_ was significantly correlated with EE_-RMP_ (EE_-IC_ = 0.295 + 0.936 × EE_-RMP_, r^2^ = 0.91). The differences between EE_-IC_ and EE_-RMP_ ranged from +0.60 to −0.54 kcal/min at rest (sitting and standing) and from +1.48 to −1.43 kcal/min for walking activities (4, 5 and 6 km/h). In their study, Gastinger et al. [15] used an equation to estimate $\dot{V}$_E-RMP_ from the four distances measured by the RMP system. After that, according to the relationship between EE and $\dot{V}$_E_, a linear regression was applied to estimate EE_-RMP_ from $\dot{V}$_E_ [13]. In our study, we directly input the four distances to the TCN model. Indeed, taking into account the perpetual variations in thoracic and abdominal dimensions with respiration, Konno and Mead [54] proposed a respiratory inductive plethysmography (RIP) method to estimate $\dot{V}$_E_ according to the movement of the chest wall and rib cage. Considering the relationship between $\dot{V}$_E_ and $\dot{V}$O_2_ ($\dot{V}$O_2_ =$\dot{\;V}$_E_ × [F_I_O_2_-F_E_O_2_]) and the relationship between $\dot{V}$O_2_ and EE (EE_-IC_ = $\dot{V}$O_2_ (L) × 4.825), we propose to directly use the changes in the thoracic and abdominal distances (RC, AB, CW, SP) to estimate EE. The differences between EE_-IC_ and EE_-RMP_ (+1.439 to −1.429 kcal/min) in adults (Figure 8 group A) are consistent with those of Gastinger et al. [15]. Furthermore, Figure 8 shows that more than 95% of the points lie within the limits of agreement intervals in the three groups. These results, therefore, appear to validate the estimation of EE directly from changes in the rib cage and abdominal distance in children, adolescents and adults. 

Comparing EE_-RMP_ and EE_-IC_ values, our results show that the RMP system seems to be effective in estimating EE, irrespective of the age of the subjects and the intensity of exercise (Table 3, Figure 6 and Figure 8). Our results even showed lower values of EE_-RMP_ for the “standing” intensity in group A (−6%) and for the “Rest-V_Th1_” intensity in group PP (−4%) and a higher value of EE_-RMP_ for the “standing” intensity in group P (+10%). These differences, even if they are significant, seem acceptable in terms of the literature. Indeed, Lopez et al. [49] used the multisensory device SenseWear Armband Mini (Body Media, Pittsburgh, PA, USA) to estimate EE in children (10.5 ± 0.7 years old), adolescents (15.3 ± 0.7 years old) and adults (37.7 ± 9.8 years old) during PAs such as sitting, walking, running, basketball and biking. They reported a slightly higher mean absolute percent error of estimate EE under sitting and standing (23–32%) compared to walking and running (8–20%). 

The overall results of our study also seem to validate the choice of a DL model to estimate EE from the RMP system. In adults, Zhu et al. [27], using a triaxial accelerometer and cardiofrequencemeter, estimated EE for walking, standing and climbing upstairs or downstairs. They evaluated the DL model convolutional neural network (CNN) and ML model artificial neural networks (ANN) in estimating EE values. Their results showed lower RMSE values with the CNN model (1.12 kcal/min) compared to the ANN model (1.73 kcal/min). Ni et al. [55] estimated EE in adults from the electrocardiogram (ECG) and inertial measurement unit (IMU) using a deep multibranch two-stage regression network (DMTRN) model during the Bruce treadmill test, carried out until exhaustion. Their results showed a close correlation between the estimated EE and the reference EE measured by an indirect calorimeter (R^2^ = 0.97, RMSE = 0.71 kcal/min). Our results (Table 2) in adults (R^2^ = 0.984, RMSE = 0.739 kcal/min) thus seem to agree with those of previous studies, such as those of Farrahi et al. [56], which show that the use of a DL model improves the accuracy of EE estimation. Using an accelerometer and a CNN model, Chowdhury et al. [48], estimated EE in eight preschool children (5.23 ± 0.75 years old) during 10 simulated free-living activities ranging in intensity from sedentary to vigorous. Their results showed that the CNN models provide efficient EE prediction (RMSE = 0.54 kcal/min). To our knowledge, our study is the first to use the TCN model to estimate EE. As a variation of the CNN model, the TCN model can extract temporal information from the data, which can improve the model’s performance [57]. Our results agree with the literature and show the interest in using a DL model to estimate EE in children, adolescents and adults under various conditions of rest and exercise intensities. Regression performance depends on the feature’s quality, which requires professional knowledge [58]. With DL models, raw sensor data can extract salient features without relying on laboriously handcrafted features [59]. However, our results show that when one intensity changes to the next intensity, the estimation error in the TCN model increases as well (Figure 7). Amelard et al. [60] indicated that the estimation error in the TCN model is likely due to the blindness of point-wise predictors to previous states. As a result, the TCN model could not learn the relationship between inputs and outputs during on-and-off transitions.

Concerning the limitations, enough datasets including various ages and anthropometric characteristics (age, sex, health, etc.) are essential for improving the accuracy of predicting EE. Indeed, it would be interesting to increase the number of subjects in each age group and also increase the age groups by adding, for example, a group of elderly people whose ventilatory responses differ from those of younger people [61]. It would also be interesting to test the RMP system on overweight or obese subjects to evaluate the ability of this system in the estimation of EE. The development of specific algorithms would then be necessary to consider the soft tissues present at the abdomen level [13]. 

## 5. Conclusions

Our results show that using a DL model and the RMP system seems efficient for estimating EE in children and adolescents under resting and exercise conditions. The findings of this study represent an essential step in the search for measurement methods and DL models for estimating EE in various subject populations. 

## Figures and Tables

**Table 1:** Pubertal stages, age and anthropometric characteristics.

Groups	*N*	Pubertal Stages (I–V)	Age (years)	Height (cm)	BM (kg)	BMI (kg/m^2^)	$\dot{V}$O_2max_ (l/min)
A.	9		28.11 ± 2.93	175.67 ± 12.98	70.66 ± 18.51	22.48 ± 2.82	3.16 ± 1.21
PP.	8	IV & V	14.75 ± 0.71	172.06 ± 7.79	56.61 ± 7.61	19.06 ± 1.12	3.29 ± 0.58
P.	6	II & III	11.67 ± 0.52	152.10 ± 4.29	41.65 ± 4.84	18.02 ± 2.29	1.99 ± 0.17

A: adult; PP: post-pubertal; P: pubertal; BM: body mass; BMI: body mass index; $\dot{V}$O_2max_: maximal oxygen uptake.

**Table 2:** TCN model performance for estimating EE and $\dot{V}$O_2_.

		EE		$\dot{V}$O_2_	
Group	Samples	R^2^	RMSE	R^2^	RMSE
	(Valid/Train)		(kcal/min)		(ml/min/kg)
A.	1590/6360	0.98	0.74	0.98	2.09
PP.	1408/5636	0.98	0.61	0.98	2.04
P.	975/3900	0.97	0.49	0.97	2.24

A: adult; PP: post-pubertal; P: pubertal; R^2^: coefficient of determination between measured EE and $\dot{V}$O_2_ and estimated EE and $\dot{V}$O_2_; RMSE: root mean square error; EE: energy expenditure; $\dot{V}$O_2_: oxygen uptake.

**Table 3:** Estimated (EE_-RMP_) and measured (EE_-IC_) energy expenditure mean values for each group and each intensity level.

Group	Intensity	EE_-IC_ ± SD	EE_-RMP_ ± SD	Mean Differences	
		Kcal/min	Kcal/min	(EE_-RMP_–EE_-IC_) ± SD	
A.	Sitting	1.19 ± 0.43	1.14 ± 0.44	−0.05 ± 0.09	NS
	Standing	1.15 ± 0.41	1.09 ± 0.40	−0.06 ± 0.08	*
	Rest-V_Th1_	4.44 ± 2.00	4.42 ± 2.00	0.06 ± 0.13	NS
	V_Th1_-V_Th2_	10.22 ± 4.15	10.28 ± 4.07	0.14 ± 0.25	NS
	$V_{\mathrm{Th}2}-\dot{V}$O_2__max_	14.64 ± 5.85	14.76 ± 5.62	0.12 ± 0.35	NS
PP.	Sitting	1.25 ± 0.20	1.25 ± 0.20	−0.01 ± 0.04	NS
	Standing	1.31 ± 0.28	1.35 ± 0.28	0.03 ± 0.04	NS
	Rest-V_Th1_	4.53 ± 1.47	4.37 ± 1.42	−0.17 ± 0.11	**
	V_Th1_-V_Th2_	10.30 ± 2.18	10.10 ± 1.96	−0.19 ± 0.30	NS
	$V_{\mathrm{Th}2}-\dot{V}$O_2__max_	15.23 ± 2.62	15.03 ± 2.41	−0.20 ± 0.40	NS
P.	Sitting	0.96 ± 0.16	0.98 ± 0.18	0.02 ± 0.06	NS
	Standing	0.99 ± 0.25	1.10 ± 0.25	0.11 ± 0.03	***
	Rest-V_Th1_	3.17 ± 0.66	3.18 ± 0.65	0.00 ± 0.09	NS
	V_Th1_-V_Th2_	6.16 ± 0.41	6.22 ± 0.32	0.06 ± 0.16	NS
	$V_{\mathrm{Th}2}-\dot{V}$O_2__max_	8.77 ± 0.87	8.90 ± 0.67	0.13 ± 0.24	NS

A: adult; PP: post-pubertal; P: pubertal; EE_-IC_: energy expenditure measured from indirect calorimetry; EE_-RMP_: energy expenditure measured from respiratory magnetometer plethysmography; V_Th1_: first ventilatory threshold; V_Th2_: second ventilatory threshold; $\dot{V}$O_2max_: maximal oxygen uptake; NS: no significant difference *p* > 0.05; *: significant difference: *p* < 0.05; **: significant difference: *p* < 0.01; ***: significant difference: *p* < 0.001.

## Data Availability

The dataset and code are available. Interested researchers could contact the corresponding authors to inquire about access.
